# Estimating the burden of care for oral potentially malignant disorders and oral cancer in Brazilian dental practice

**DOI:** 10.4317/medoral.26701

**Published:** 2024-08-18

**Authors:** João Paulo Gonçalves de Paiva, Jacks Jorge, Erison Santana dos Santos, Arn Migowski, Daniel Cohen-Goldemberg, Luiz Paulo Kowalski, Thaís Bianca Brandão, Ana Carolina Prado Ribeiro, Marcio Ajudarte Lopes, Pablo Agustin Vargas, Eurípides Alves da Silva, Saman Warnakulasuriya, Alan Roger Santos-Silva

**Affiliations:** 1 DDS, MSc. Department of Oral Diagnosis, Piracicaba School of Dentistry, State University of Campinas, Piracicaba, São Paulo, Brazil; 2 DDS, PhD. Department of Oral Diagnosis, Piracicaba School of Dentistry, State University of Campinas, Piracicaba, São Paulo, Brazil; 3 MD, PhD. Clinical Research and Technological Development Division, Research and Innovation Coordination, National Cancer Institute (INCA), Ministry of Health, Rio de Janeiro, Brazil. Professional Master’s Program in Health Technology Assessment, Teaching and Research Coordination, Instituto Nacional de Cardiologia (INC), Ministry of Health, Rio de Janeiro, Brazil; 4 DDS, PhD. Clinical Research Division, National Cancer Institute of Brazil (INCA), Rio de Janeiro, Brazil. Honorary Associate Professor of Oral Medicine, Eastman Dental Institute, University College London, UK; 5 MD, PhD. Head and Neck Surgery Department, Institute of Cancer of São Paulo, University of São Paulo Medical School, São Paulo, Brazil; 6 DDS, PhD. Serviço de Odontologia Oncológica, Institute of Cancer of São Paulo, University of São Paulo Medical School, São Paulo, Brazil; 7 DDS, PhD. Hospital Sírio Libanês, São Paulo, Brazil; 8 Bmath, PhD. Instituto de Biociências, Letras e Ciências Exatas, UNESP - São Paulo State University, São José do Rio Preto, São Paulo, Brazil; 9 Emeritus Professor. Faculty of Dentistry, Oral and Craniofacial Sciences, King's College London, UK; WHO Collaborating Centre for Oral Cancer, London, UK

## Abstract

**Background:**

Oral cancer (OC) and oral potentially malignant disorders (OPMD) pose significant challenges to public health in Brazil. This study aimed to estimate the prevalence of oral cancer (OC) and oral potentially malignant disorders (OPMD) among patients who would be treated by Brazilian dentists during their careers.

**Material and Methods:**

Data on the number of dentists in Brazil were extracted from the 2022 census data, while incidence rates for OC cases were sourced from the Brazilian National Cancer Institute (INCA). Population estimates for Brazil and data on dental check-up rates were obtained from relevant national sources.

**Results:**

Our analysis indicates that a general dentist in Brazil can expect to encounter on average two to three OC patients and on average 675 patients with OPMDs over a 35-year career. Regional disparities were observed, with certain regions showing higher than the average number of encounters due to low density of dentists in some rural districts.

**Conclusions:**

Brazilian dentists are likely to confront a substantial number of OPMD cases during their professional tenure, emphasizing the need for public health policies aimed at enhancing dental surgeons' education in oral cancer prevention and early detection.

** Key words:**Oral cancer, potentially malignant disorders, dentistry, public health, burden estimation.

## Introduction

Oral cancer (OC) and oral potentially malignant disorders (OPMD) pose significant public health challenges, particularly in certain geographic areas in Brazil (1). According to the Brazilian National Cancer Institute (INCA) for the years 2023-2025, Brazil is expected to annually diagnose 15,100 new patients of OC, ranking OC as the eighth most common cancer among the Brazilian population (the fifth most common among men) (https://www.gov.br/inca/pt-br/assuntos/cancer/numeros/estimativa) (accessed in May 2023). Despite Brazil recording the highest number of dentists globally, a significant issue persists with late diagnoses of most OC patients in the country (2-4).

Approximately 48% of the Brazilian population routinely seeks dental consultations, a behavior that is closely linked to the early detection of OC and OPMD (5). With an estimated prevalence of 3.93% for OPMD within the Brazilian population, it becomes evident that Brazil encounters a substantial number of OPMD patients, primarily due to its large population, but also because the population is exposed to social inequalities, low levels of health literacy, and tobacco and alcohol use with an estimated prevalence of 3.93% for OPMD within the Brazilian population, it becomes evident that Brazil encounters a substantial number of OPMD patients, primarily due to its large population, but also because the population is exposed to social inequalities, low levels of health literacy, and tobacco and alcohol use.” (6). This underscores the pivotal role that Brazilian dentists could play, not only in OC diagnosis but also in the management of OPMD. There are sound opportunities for the provision of crucial information to patients regarding health education, associated risk factors, disease progression, treatment, and educating on early signs and symptoms of OC and in screening for OPMDs during routine oral examinations (3,7,8).

Analyzing this paradoxical scenario marked by a massive dental workforce operating in a territory characterized by high mortality rates due to OC, raises questions about the potential of each professional to identify, diagnose, and provide appropriate and rapid treatment for patients with OPMDs and OCs throughout their careers. Therefore, influenced by a previous study carried out in the UK (9), the current study was undertaken as an exploratory analysis with the aim of elucidating the estimated number of patients with OC and OPMDs who will potentially cross the professional paths of Brazilian dentists over the course of a 35-year career.

## Material and Methods

To estimate the Brazilian population, we relied on the 2022 Brazilian demographic census results (https://censo2022.ibge.gov.br/panorama/) (accessed in May 2023). Data on the total count of active Brazilian dentists, encompassing specialists in stomatology (oral medicine), oral and maxillofacial surgery, oral and maxillofacial pathology, and general dentists were obtained from records accessible on the Brazilian Federal Dental Council website (https://cfo.org.br/website/estatisticas/quantidade-geral-de-entidades-e-profissionais-ativos/) (accessed in May 2023). We considered a 35-year period as the standard career duration for a Brazilian dentist. The estimated number of Brazilians who regularly sought dental consultations as well as the ratio of Brazilian dentists per 2,000 inhabitants were based on the findings of the 2019 National Health Survey (5).

**Figure 1:**
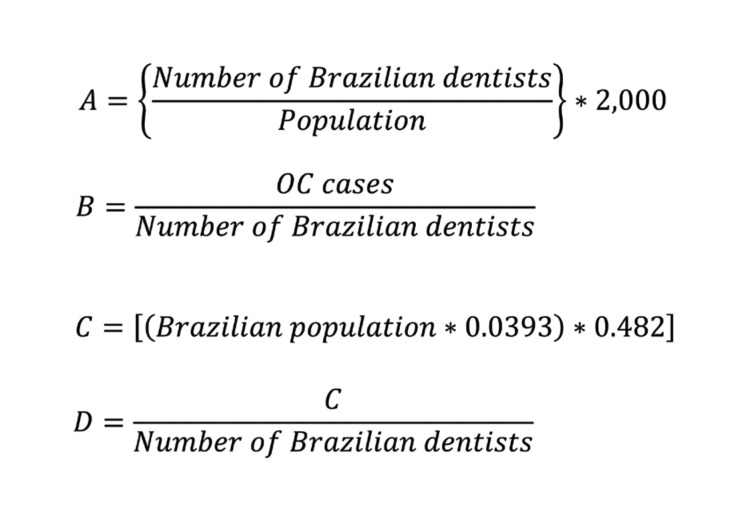
(A) Mathematical equation used to define the ratio of inhabitants per Brazilian dentist. (B) Mathematical equation utilized to estimate the number of OC possible diagnoses per Brazilian dentist. (C) Mathematical equation used to estimate the number of possible OPMD patients who seek dental consultations annually. (D) Mathematical equation utilized to determine the number of possible OPMD diagnoses per Brazilian dentist.

The dentist-to-resident population ratio within a given region was determined using the mathematical equation displayed in Fig. 1.

The estimated OC cases within the Brazilian population were derived from data provided by INCA for the years 2023-2025 (https://www.gov.br/inca/pt-br/assuntos/cancer/numeros/estimativa) (accessed in May 2023). In INCA statistics, OC encompasses malignant diseases affecting various anatomical sites, each represented by their respective codes: lip (C00), tongue base (C01), other tongue regions (C02), gingiva (C03), floor of the mouth (C04), palate (C05), other mouth regions (C06), parotid gland (C07), other major salivary glands (C08), tonsil (C09), and oropharynx (C10) (https://www.gov.br/inca/pt-br/assuntos/cancer/numeros/estimativa) (accessed in May 2023). The estimated cases of OPMD within the Brazilian population were determined based on the results of a prior systematic review that established the global prevalence of these conditions (6). We adopted a prevalence rate of 3.93% for OPMD in South America and the Caribbean, as these data are considered more representative for the Brazilian population than individual studies, as 62.5% of the studies used in this meta-analysis to estimate this prevalence for South America and the Caribbean were carried out in Brazil. Therefore, Brazil was the country that was best represented in this estimate and with the greatest influence on the estimated prevalence for the region (6). To ascertain the estimated annual number of OC patients that a Brazilian dentist would diagnose per year, the mathematical equation displayed in Fig. 1 was used.

We assumed that all OC patients in Brazil would seek dental consultation at least once during the natural history of the disease, as INCA statistics rely on histopathologically confirmed OC cases, and most of these diagnoses were defined by dentists.

To estimate the OPMD patients who had sought dental care at least once in the past year, the mathematical equation displayed in Fig. 1 was used.

To ascertain the estimated annual number of OPMD patients that a Brazilian dentist would diagnose per year, the mathematical equation demonstrated in Fig. 1 was utilized.

To estimate the total number of OC and OPMD patients that a Brazilian dentist would potentially diagnose throughout an entire 35-year career, the results obtained from the equations B and D were multiplied by 35. These estimations were applied uniformly across the entire country, encompassing its federal states and capital cities.

## Results

The 2023 Brazilian demographic census revealed a total estimated population of 203,062,512 citizens (https://censo2022.ibge.gov.br/panorama/) (accessed in May 2023). Within this population, approximately 48.2% sought dental care at least once annually, accounting for 97,863,220.38 patients (5). As of May 2023, the Brazilian Federal Dental Council website reported approximately 393,299 actively practicing dentists in Brazil. Most of these dentists were stationed in the southeastern and southern regions, with the highest numbers in São Paulo (*n*=111,025/ 28.23%), Minas Gerais (*n*=47,148/ 11.99%), Rio de Janeiro (*n*=36,288/ 9.23%), and Paraná (*n*=28,813/ 7.33%) states (Fig. 1). All Brazilian federal states, along with their respective capitals, exceeded the recommendation of one dentist per 2,000 inhabitants established by the Brazilian Federal Dental Council according to resolution number 1,631 published in October 2015 (https://bvsms.saude.gov.br/bvs/saudelegis/gm/2015/prt1631_01_10_2015.html) (accessed in May 2023). The highest proportions were observed in Paraná (*n*=5.04), São Paulo (*n*=5), Minas Gerais (*n*=4.59), and Rio de Janeiro states (*n*=4.52). In the capitals, the highest proportions per 2000 inhabitants were found in Vitória (*n*=12.85), Florianópolis (*n*=9.48), Belo Horizonte (*n*=8.71), and Curitiba (*n*=8.59) (Table 1). From all the recognized dental specialties in Brazil, we selected those with competencies related to the diagnosis of OC and OPMD, in accordance with Brazilian Federal Dental Council Resolution 63/2005. The dental specialties in Brazil commonly involved in diagnosing OC and OPMD include oral and maxillofacial surgery (*n*=7,011/ 1.78% of all Brazilian dentists), stomatology (oral medicine) (*n*=1,061/ 0.26%), and oral and maxillofacial pathology (*n*=422/ 0.10%). General dentists constitute approximately 199,057 professionals, accounting for 50.61% of the total (Table 2).

As per data provided by INCA for the years 2023 to 2025, Brazil is anticipated to diagnose approximately 15,100 OC cases annually (https://www.gov.br/inca/pt-br/assuntos/cancer/numeros/estimativa) (accessed in May 2023). When we calculate the possible number of OC patients visiting Brazilian dentists, we ascertain that each Brazilian dentist may encounter approximately 0.04 OC patients each year. Assuming a consistent trend over the years, the national average for overall dentists is approximately 1.34 OC cases during a 35-year career.

**Figure 2:**
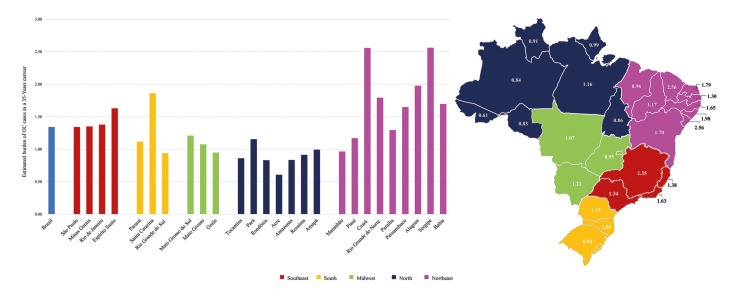
Comparative average potential OC diagnosis across Brazilian federal states.

An in-depth analysis of each federal state and its respective capital city unveils regional disparities, with certain areas, such as Ceará (*n*=2.56) and Sergipe (*n*=2.56), displaying a nearly twofold higher likelihood of encountering OC patients throughout their careers compared to dentists in other regions. States such as Alagoas (*n*=1.98), Santa Catarina (*n*=1.86), and Rio Grande do Norte (*n*=1.79) exhibit elevated rates across the country, while Acre (*n*=0.61), Amazonas (*n*=0.84), and Rondônia (*n*=0.83) show comparatively lower rates. Examining the capital cities, some, such as Fortaleza (*n*=1.33) and Maceió (*n*=1.23), present elevated rates of potential OC diagnoses compared to the national average for overall dentists (Table 1, Fig. 2).

Based on an estimated OPMD prevalence of 3.93% in South America, Brazil is projected to harbor roughly 7,980,356 OPMD patients. Of this patient population, approximately 3,846,531 seek dental care annually. Within a single year, the national average for overall dentists comprises 9.78 OPMD cases. Over the course of their career, dentists can collectively diagnose approximately 342 OPMD cases. Notably, certain Brazilian federal states, including Maranhão (*n*=687), Pará (*n*=683), and Ceará (*n*=560), exhibit the highest numbers of diagnosable OPMD cases. Conversely, the lowest rates of anticipated OPMD diagnosis are observed in Paraná (*n*=263), São Paulo (*n*=265), and Minas Gerais (*n*=288). Importantly, the average of OPMD possible diagnoses over a dentist’s career in many federal states surpasses the national average for overall dentists (Table 3). In federal states, dentists would potentially be more likely to diagnose OPMD cases over their professional careers due to the lower concentration of professionals (Table 3).

Across a 35-year career, oral and maxillofacial pathologists are expected to diagnose approximately 1,252.37 cases of OC and 319,025 cases of OPMD. Stomatology (oral medicine) professionals are expected to diagnose 498 OC cases and 126,888 OPMD cases, while oral and maxillofacial surgeons are projected to diagnose 75 OC cases and 19,202 OPMD cases. In contrast, General dentists (professionals without registered specialty) are estimated to diagnose 2.66 OC cases and a significant number of 676 OPMD cases (Table 2).

## Discussion

This study represents a novel approach in the Pan-American context, representing the first attempt to estimate the burden of OPMD and OC cases that potentially fall within the care of Brazilian dental surgeons who are generally responsible for the diagnosis of both these conditions. Despite its descriptive nature, our findings highlight evident discrepancies in the diagnostic opportunities for OPMD and OC cases among dentists across the country. This initial analysis can contribute to understanding the paradoxical scenario regarding oral cancer (OC) in Brazil, where a high number of dentists coexist with a high prevalence of OC cases, which are often diagnosed at advanced stages. It highlights the pivotal role of this workforce in Brazil (2-4).

Some Brazilian regions are characterized by a higher number of OC incident cases as well as deaths due to the disease, such as the Southeastern and Southern regions (10). However, in other areas of Brazil, particularly in the Northeast, the incidence and mortality rates might be underestimated, due to a significant number of deaths being recorded without registering specified causes of mortality (1). The Southern state of Santa Catarina stands out with a notably higher rate (*n*=1.86) of potential OC diagnoses. The Santa Catarina state has the lowest dentist-to-inhabitant ratio among the states in these two regions and was determined as the region with the most efficient workflow processes for OC diagnosis and treatment in Brazil (11). These factors facilitate access to OC diagnosis for patients and could explain the high number of possible OC diagnoses per dentist in this region (11).

The federal states that recorded the highest number of potential OC diagnoses per dentist were in the Northeastern region of Brazil, such as Sergipe and Ceará. The Northeastern region of Brazil is the second highest in the country in terms of smoking prevalence, which is an established risk factor for OC development (12). On the other hand, the states of Sergipe and Ceará have some of the lowest dentist-to-inhabitant ratios in the northeastern region. The combination of high smoking rates and a shortage of dentists in these two states may explain the high demand for stomatological care in the region. Furthermore, the Northeastern region experiences significant sunlight exposure, which is recognized as a risk factor for lip cancer development (13).

Among the Brazilian capital cities, Fortaleza had the highest number of possible oral cancer cases per dentist, compared to the national average. Studies on the challenges associated with diagnosing OC in Fortaleza city revealed that professionals working in the public health system, despite possessing the technical knowledge required for diagnostic procedures, encounter difficulties linked to prioritizing outpatient dental care in public health services, and shortage of technical equipment necessary for conducting surgical procedures (14). Similar challenges are evident across all levels of healthcare in Brazil, which have a direct impact as barriers to the reduction of the OC incidence and mortality rates in the country (1,2). Dental secondary care in Brazil is provided through the work of the Dental Specialties Centers (acronym in Portuguese, CEO), which include stomatology (oral medicine) professionals primarily focusing on diagnosing OC and other stomatological diseases. Within this framework, Brazil experiences divergent patterns in secondary healthcare, marked by an increase in continuing education for professionals in these services alongside a decrease of about 17.8% in CEOs qualified for performing biopsies and an 18.3% reduction in access to hospitals for referring diagnosed OC patients to treatment - a necessary step in primary care to improve the 5-year survival rate of this disease (11,15-17).

There have been limited studies conducted to investigate the prevalence of OPMD in the Brazilian population, with noteworthy research originating from the Northeastern and Southeastern regions (18-20). In the future, if population-based surveys were to be carried out, these estimates could be improved, since there is a dearth of epidemiological studies reporting OPMD prevalence (6). A survey carried out in Indonesia, a country that is not considered to have a high OC incidence, demonstrated a prevalence of OPDM many times higher than used in the present study (14.1%) (21).

In Brazil, the diagnosis of OC and OPMD is usually conducted by specific dental specialties or by dentists working within the Brazilian Unified Health System (acronym in Portuguese, SUS) at the primary or secondary care levels (1,22). Stomatology (oral medicine) and oral and maxillofacial pathology specialists are the only professionals who have access to a comprehensive curriculum focused on the diagnosis of oral and maxillofacial diseases. However, all Brazilian dentists receive minimal training in diagnostic skills during their undergraduate courses (15).

As shown in Table 2, our results underscore the significant contribution of oral and maxillofacial pathology and stomatology (oral medicine) professionals in the diagnosis of OC and OPMD in Brazil (Table 2). These dental specialties demonstrated the highest potential diagnoses over the course of a Brazilian dentist career. Brazil harbors influential oral and maxillofacial pathology and stomatology (oral medicine) experts around the world, most of them based in the Southeast region. The unequal distribution of these experts across the country might contribute to the difficult access for OPMD and OC patients seeking care, emphasizing the need to decentralize the coverage of oral diagnostic professionals across the country (22).

The incidence of OC in Brazil is noteworthy, particularly among individuals with lower socioeconomic status who often rely on public healthcare services for diagnosis and treatment (1). Given this context, it is pertinent to recognize the role of general dentists, who constitute a significant portion of the dental workforce in Brazil and are actively engaged in public healthcare initiatives, contributing to the early detection and management of OC and OPMD patients (16,23). Against this backdrop, the findings of our study shed light on the substantial burden potentially faced by Brazilian general dentists in terms of OC and OPMD diagnoses over their professional lifespan revealing that a Brazilian general dentist can expect to encounter an average of two to three OC and 675 OPMD cases over a 35-year career. Seeing two cancer cases in a career has been established as a norm in a similar study conducted in the UK (9). Another study conducted in Scotland showed that Scottish dentists can encounter 0.01 OC cases annually (24). Furthermore, although our study did not stratify the results by whether or not dentists worked in the SUS, general dentists who serve the SUS population likely have a greater demand for OPMD and OC throughout their careers than dentists who treat patients in private offices (Table 4).

Despite the valuable insights gained from our study, certain limitations were noted. These include the utilization of data from various timeframes, the absence of official statistics from entities such as the Brazilian Dental Council (CFO) and dental specialty associations, and the inability to adjust OC incidence rates over the span of a Brazilian dentist’s professional career. Additionally, our analysis revealed a bias in the systematic review utilized to estimate the potential diagnoses OPMD per dentist because, notably, the primary studies incorporated in this review, which were focused on Brazilian patients, relied predominantly on laboratory registers, potentially skewing the true prevalence of OPMD in Brazil. However, it's worth considering that some OC and OPMD patients may initially consult a family physician who then refers them to specialists. This process could potentially reduce the number of diagnostic opportunities for OC and OPMD per Brazilian dentist (25). Moreover, considering projections from the Global Cancer Observatory indicating a steady increase in the incidence and mortality of OC until 2040, it is conceivable that Brazilian dentists, in the future, will encounter a greater number of opportunities to diagnose OC throughout their careers than initially anticipated in our study (26).

Considering the findings elucidated by this study, it is essential to enact health policy-driven strategies aimed at strengthening the curriculum for undergraduate dental students throughout Brazil. Emphasis should be placed on the evolving landscape of OC epidemiology, fostering a comprehensive understanding of OPMD and OC, and disseminating information to dentists regarding the prompt referral, diagnostic, and treatment protocols for patients presenting with OC or OPMD (23). Furthermore, it is imperative to allocate resources towards enhancing the training and technical capabilities of dentists practicing in both primary and secondary healthcare settings. This includes refining the processes involved in patient referrals across various tiers of healthcare provision and improving secondary databases such as the population-based cancer registries and the SUS outpatient information system (11,16,27). These concerted efforts are crucial steps toward improving early detection, diagnosis, and management of OC and OPMD cases, ultimately contributing to better outcomes for affected individuals within the Brazilian population.

## Conclusions

In conclusion, in some Brazilian federal states and capital cities, dentists are more likely to diagnose OC and OPMD patients, such as in Ceará, Sergipe, Maranhão, Fortaleza, Rio Branco, and Porto Velho. Brazilian general dentists might face more than two OCs cases and 600 OPMDs over their careers. Brazilian oral and maxillofacial pathologists as well as stomatologists (oral medicine) are the specialists with the largest possibility of diagnosing OC and OPMD nationwide. There is an urgent need for investments aimed at enhancing the technical training and diagnostic skills of both undergraduate dental students and for continuing professional education to active dentists working in Brazil.

## Figures and Tables

**Table 1:** Geographical variation in dentist density and OC metrics across the Brazilian Federal States and their respective capital cities.

Federal State		Dentists (n)	Dentists per 2,000 inhabitants (n)	OC cases (n)	OC patients in a 35-year career (n)	Capital city	Dentists (n)	Dentists per 2,000 inhabitants (n)	OC cases (n)	OC patients in a 35-year career (n)
Brazil		393,299	3.87	15,100	1.34	-	-	-	-	-
Southeast	São Paulo	111,025	5.00	4,260	1.34	São Paulo	37,079	6.48	1,020	0.96
	Minas Gerais	47,148	4.59	1,820	1.35	Belo Horizonte	10,084	8.71	290	1.01
	Rio de Janeiro	36,288	4.52	1,430	1.38	Rio de Janeiro	17,744	5.71	570	1.12
	Espírito Santo	7,732	4.03	360	1.63	Vitória	2,075	12.85	30	0.51
South	Paraná	28,813	5.04	920	1.12	Curitiba	7,621	8.59	140	0.64
	Santa Catarina	12,217	3.21	650	1.86	Florianópolis	2,546	9.48	60	0.82
	Rio Grande do Sul	21,608	3.97	580	0.94	Porto Alegre	5,316	7.98	90	0.59
Midwest	Mato Grosso do Sul	5,210	3.78	180	1.21	Campo Grande	2,342	5.22	70	1.05
	Mato Grosso	6,838	3.74	210	1.07	Cuiabá	2,043	6.28	50	0.86
	Goiás	14,382	4.08	390	0.95	Goiânia	5,728	7.97	130	0.79
North	Tocantins	2,843	3.76	70	0.86	Palmas	948	6.26	20	0.74
	Pará	7,875	1.94	260	1.16	Belém	3,808	5.84	90	0.83
	Rondônia	2,945	3.73	70	0.83	Porto Velho	1,123	4.88	20	0.62
	Acre	1,153	2.78	20	0.61	Rio Branco	958	5.25	20	0.73
	Amazonas	5,865	2.98	140	0.84	Manaus	5,267	5.10	100	0.66
	Roraima	1,148	3.52	30	0.91	Boa Vista	1,055	5.10	20	0.66
	Amapá	1,410	3.84	40	0.99	Macapá	1,189	5.37	30	0.88
Northeast	Maranhão	6,531	1.93	180	0.96	São Luís	3,028	5.84	50	0.58
	Piauí	4,191	2.56	140	1.17	Teresina	2,668	6.16	40	0.52
	Ceará	10,396	2.36	760	2.56	Fortaleza	6,043	4.98	230	1.33
	Rio Grande do Norte	5,073	3.07	260	1.79	Natal	2,669	7.11	70	0.92
	Paraíba	6,755	3.40	250	1.30	João Pessoa	3,151	7.56	50	0.56
	Pernambuco	12,312	2.72	580	1.65	Recife	5,247	7.05	120	0.80
	Alagoas	4,068	2.60	230	1.98	Maceió	2,849	5.95	100	1.23
	Sergipe	2,731	2.47	200	2.56	Aracajú	2,113	7.01	40	0.66
	Bahia	18,569	2.63	900	1.70	Salvador	6,543	5.41	210	1.12

**Table 2:** Annual and career-long diagnostic capacities of dental specialists for OC and OPMD in Brazil.

Specialty	Specialists (n)	OC cases per specialist annually (n)	OC cases over a 35-year career (n)	OPMD cases per specialist annually (n)	OPMD cases over a 35-year career (n)
Oral and maxillofacial pathology	422	35.78	1,252.37	9,115	319,025.10
Stomatology (oral medicine)	1,061	14.23	498.11	3,625.38	126,888.42
Oral and maxillofacial surgery	7,011	2.15	75.38	548.64	19,202.48
General dentists	199,057	0.08	2.66	19.32	676.33

**Table 3:** Estimated OPMD prevalent cases and dentist diagnostic potential across Brazilian federal states and their respective capital cities.

Federal State		Estimated OPMD cases (n)	Estimated OPMD cases seen by a dentist (n)	OPMD cases seen by each dentist annually (n)	OPMD cases in a 35-year career (n)	Capital city	Estimated OPMD cases (n)	Estimated OPMD cases seen by a dentist (n)	OPMD cases seen by each dentist annually (n)	OPMD cases in a 35-year career (n)
Brazil		7,980,356.72	3,846,531.94	9.78	342.31	-	-	-	-	-
Southeast	São Paulo	1,745,724.04	841,438.99	7.58	265.26	São Paulo	450,033.93	216,916.35	5.88	205.71
	Minas Gerais	807,171.62	389,056.72	8.25	288.81	Belo Horizonte	91,001.51	43,862.73	4.44	155.46
	Rio de Janeiro	630,942.79	304,114.43	8.38	293.32	Rio de Janeiro	244,108.92	117,660.50	6.66	233.12
	Espírito Santo	150,656	72,616.19	9.39	328.71	Vitória	12,688.75	6,115.98	2.94	102.91
South	Paraná	449,718.07	216,764.11	7.52	263.31	Curitiba	69,707.71	33,599.11	4.47	156.34
	Santa Catarina	299,057.32	144,145.63	11.80	412.96	Florianópolis	21,112.47	10,176.21	4.13	144.55
	Rio Grande do Sul	427,603.89	206,105.07	9.54	333.84	Porto Alegre	52,370.00	25,242.34	4.80	167.90
Midwest	Mato Grosso do Sul	108,338.31	52,219.07	10.02	350.80	Campo Grande	35,288.96	17,009.28	7.36	257.72
	Mato Grosso	143,791.35	69,307.43	10.14	354.75	Cuiabá	25,580.84	12,329.97	6.02	210.72
	Goiás	277,270.46	133,644.36	9.29	325.24	Goiânia	56,483.41	27,225.01	4.80	168.09
North	Tocantins	59,400.34	28,630.96	10.07	352.47	Palmas	11,895.80	5,733.77	6.15	215.32
	Pará	318,963.99	153,740.64	19.52	683.29	Belém	51,223.19	24,689.58	6.54	228.91
	Rondônia	62,133.93	29,948.55	10.17	355.93	Porto Velho	18,094.23	8,721.42	7.79	272.79
	Acre	32,620.02	15,722.85	13.64	477.28	Rio Branco	14,334.91	6,909.43	8.19	286.53
	Amazonas	154,888.18	74,656.10	12.73	445.52	Manaus	81,097.40	39,088.95	7.64	267.42
	Roraima	25,651.62	12,364.08	10.77	376.95	Boa Vista	16,250.00	7,832.50	7.46	261.08
	Amapá	28,826.86	13,894.55	9.85	344.90	Macapá	17,407.27	8,390.30	7.21	252.29
Northeast	Maranhão	266,263.47	128,338.99	19.65	687.78	São Luís	40,784.56	19,658.16	6.59	230.57
	Piauí	128,479.56	61,927.15	14.78	517.17	Teresina	34,045.59	16,409.97	6.40	224.09
	Ceará	345,513.34	166,537.43	16.02	560.68	Fortaleza	95,447.05	46,005.48	7.81	273.33
	Rio Grande do Norte	129,784.56	62,556.16	12.33	431.59	Natal	29,526.09	14,231.58	5.35	187.33
	Paraíba	126,197.65	75,287.27	11.15	390.09	João Pessoa	32,773.53	15,796.84	5.17	181.04
	Pernambuco	355,985.49	171,585.01	13.94	487.77	Recife	58,514.56	28,204.02	5.45	190.60
	Alagoas	122,911.18	59,243.19	14.56	509.71	Maceió	37,646.10	18,145.42	6.42	224.73
	Sergipe	86,835.63	41,854.77	15.33	536.40	Aracajú	23,688.35	11,417.78	5.60	195.99
	Bahia	555,561.19	267,780.49	14.42	504.73	Salvador	95,027.60	45,803.30	7.01	245.27

**Table 4:** The burden of care for OC patients in previous studies.

Author/Year	Country	Oral cancer cases per dentist annually
Ogden *et al.* (9)	United Kingdom	0.01
Purkayastha *et al.* (24)	Scotland	0.1
Paiva et al.*	Brazil	0.04

*Current study.
